# Elimination of primordial germ cells in sturgeon embryos by ultraviolet irradiation^†^

**DOI:** 10.1093/biolre/ioy076

**Published:** 2018-04-07

**Authors:** Taiju Saito, Hilal Güralp, Viktoriia Iegorova, Marek Rodina, Martin Pšenička

**Affiliations:** Research Institute of Fish Culture and Hydrobiology, South Bohemian Research Center of Aquaculture and Biodiversity of Hydrocenoses, Faculty of Fisheries and Protection of Waters, University of South Bohemia in Ceske Budejovice, Vodnany, Czech Republic

**Keywords:** primordial germ cells, developmental biology, early development, embryo, fish reproduction

## Abstract

A technique for rescuing and propagating endangered species involves implanting germ line stem cells into surrogates of a host species whose primordial germ cells (PGCs) have been destroyed. We induced sterilization in sterlet (*Acipenser ruthenus*) embryos by means of ultraviolet (UV) irradiation at the vegetal pole, the source of early-stage PGCs of sturgeon eggs. The optimal cell stage and length of UV irradiation for the effective repression of the developing PGCs were determined by exposing embryos at the one- to four-cell stage to different doses of irradiation at a wavelength of 254 nm (the optimal absorbance spectrum for germplasm destruction). The vegetal pole region of the embryos was labeled immediately upon irradiation with GFP *bucky ball* mRNA to monitor the amount of germ plasm and FITC-dextran (M.W. 500,000) to obtain the number of PGCs in the embryos. The size of the germ plasm and number of surrounding mitochondria in the irradiated embryos and controls were observed using transmission electron microscopy, which revealed a drastic reduction in both on the surface of the vegetal pole in the treated embryos. Furthermore, the reduction in the number of PGCs was proportional to the dose of UV irradiation. Under the conditions tested, optimum irradiation for PGCs removal was seen at 360 mJ/cm^2^ at the one-cell stage. Although some PGCs were observed after the UV irradiation, they significantly reduced in number as the embryos grew. We conclude that UV irradiation is a useful and efficient technique to induce sterility in surrogate sturgeons.

## Teaser

Sturgeon primordial germ cells can be terminated instantly by UV irradiation (254 nm) at the one-cell stage.

## Introduction

Sterilization is becoming an important component of the package of practices in fish culture for a variety of reasons. A major motivation is the minimization of the potentially deleterious ecological impact from escaping fish, while simultaneously protecting valuable strains from unauthorized propagation [1]. In addition, it facilitates the technique of germ cell transplantation (GCT), by which germ line stem cells (GSCs) of the target species are introduced into sterilized recipients of a surrogate species. GCT has been shown to be a powerful technique for obtaining gametes efficiently via the germline chimera and thus boosting fish seed production in several fish species. There have been many examples of its use in the past decade (reviewed by [2]). Surrogate sterilization ensures the exclusive production of donor-derived gametes, especially useful when rescuing endangered target species.

Sturgeons (family Acipenseridae) have been declared by IUCN (International Union for Conservation of Nature) as “more critically endangered than any other group of species,” with 27 species making the “Red List” (IUCN. 18 March 2010). Therefore, these species are ideally positioned to take advantage of the surrogate production technique (GCT) and be rescued. Previous studies from our lab have already reported techniques to enrich GSCs from enzymatically digested ovarian or testicular cells by using the Percoll density gradient approach [3]. We showed that enriched sturgeon cells colonized successfully at the genital ridge of recipients after being transplanted into the body cavity, a result also shown for other species (reviewed by [2,4,5]). Our lab has focused on the sterlet sturgeon (*Acipenser ruthenus*), the smallest (less than 120 cm) and fastest reproducing (within five years) species in Acipenseriformes, as the host or surrogate species. Transplanting germline stem cells from target sturgeon species with large body size and long reproduction cycle, such as beluga sturgeon (up to 7 m in body length and more than 20 years before the first spawning) into sterlet hosts, can dramatically reduce the space and time required for gamete production. This technique, along with cryopreservation of sturgeon GSC (the only way, at the present time, to preserve both maternal and paternal genetic information simultaneously [6]) should also go a long way in also bringing economic benefits to the fish farmer.

One limiting factor of the surrogate reproduction technique in sturgeons is the sterilization of the surrogate sturgeon species. Although the injection of antisense morpholino oligonucleotide (MO) against the *dead end* gene into fertilized eggs eliminates primordial germ cells (PGCs) in sterlet embryos [7], mass production of sterilized recipients using this system is still very labor-intensive. Furthermore, since the technique uses microinjection, it requires specialized training in the delicate handling of the eggs with forceps, and the use of a micromanipulator, microinjector, and micro glass needle under a microscope. In addition, the microinjection needs to be done quickly (within the early cleavage stages), and the MO reagent is expensive and toxic to the eggs even at slightly higher doses.

Techniques used to achieve sterilization in some species for recipient production do not work well in sturgeons. For example, triploids are used as sterile recipients for surrogate production in goldfish, trout, nibe croaker, etc. This technique is useful only if conditions for temperature or pressure shock have been optimized for the production of triploids, which are then confirmed to be free of functional germ cells. It is not clear what these optimal conditions are or even if sterility is achieved in sturgeons. Recently, a spontaneous hexaploid (1.5-fold increment in number of chromosome sets) male Siberian sturgeon was reported in a hatchery stock and it was revealed that the individual was fully fertile [8]. Such fertility in the chimeric host (after GSC transplantation) can lead to genetic contamination, making it expensive to monitor and difficult to sell or release the fish.

In some fish species, sterilization by busulfan treatment has been reported (reviewed by [9]). Injection of busulfan, originally a cancer drug, into the muscle or the peritoneal cavity, combined with high temperature treatment, causes death of rapidly proliferating cells. It can be used only in warmwater fish species (such as tilapia), where the proliferation of spermatogonia is initiated by increasing the temperature. However, in sturgeons the spermatogonia proliferation begins when the temperature decreases (in autumn) [10], thus making busulfan unsuitable for use on sturgeons. Furthermore, busulfan treatment is known to cause side effects in fish, and the efficiency of GSCs removal is not always perfect [11–13]. Finally, sturgeons, being relatively large coldwater fish, would require a relatively large amount of busulfan and long-term treatment before the fish are able to eliminate all their endogenous germ cells. Thus, busulfan treatment does not appear to be applicable for sturgeons.

The development of sturgeon PGCs is similar to that of anurans in that they are specified by the inheritance of maternally supplied germ plasm at the peripheral layer of the vegetal pole in the early-stage embryo [14]. The germ plasm in anurans is in the form of a dense “nuage,” which is composed of various proteins, mRNAs, and mitochondoria [15], and has been shown to be fragile and sensitive to UV radiation [16,17]. Inspired by this latter fact, we set out in this study to determine if sterilization by UV irradiation is applicable to sturgeons as well, since disruption of the germ plasm association/development by UV irradiation would permit a large-scale production of sterilized recipients. We tested this using sterlet embryos.

## Materials and methods

### Ethics

All experimental procedures were performed in accordance with national and institutional guidelines on animal experimentation and care, and were approved by the Animal Research Committee of University of South Bohemia, Ceske Budejovice, Czech Republic.

### Experimental design

**Figure 1.**
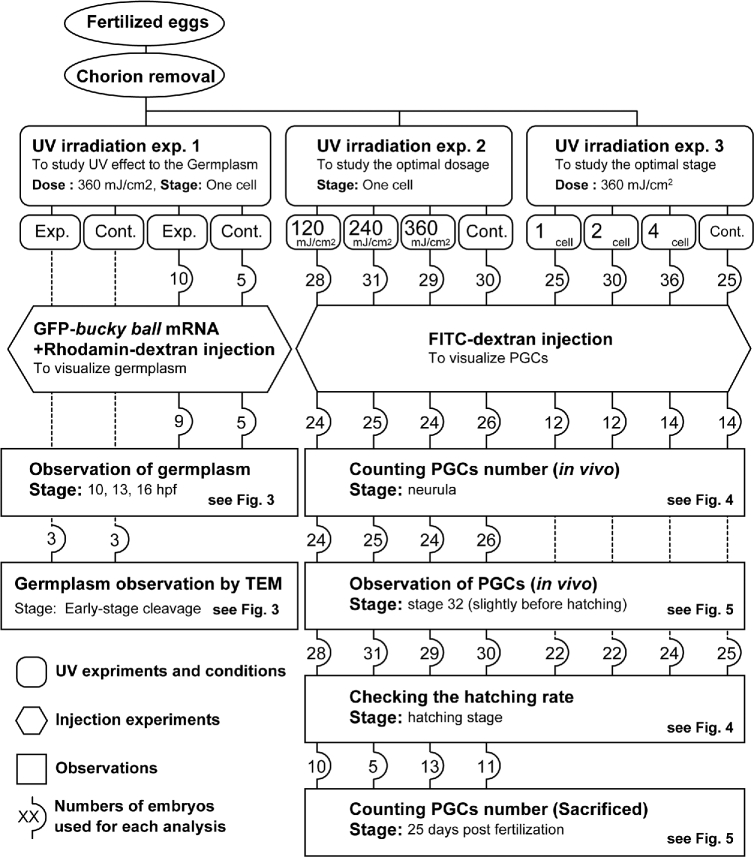
A flow diagram of this study. Exp.: Experiment; Cont.: Control.

The experiments comprised UV irradiation and microinjection of mRNA or tracer dye, and the results were obtained by the visualization of the germ plasm or PGCs (Figure 1). The sample size (number of embryos) used in each analysis is shown within rounded squares in the figure.

### Preparation of embryos

**Figure 2.**
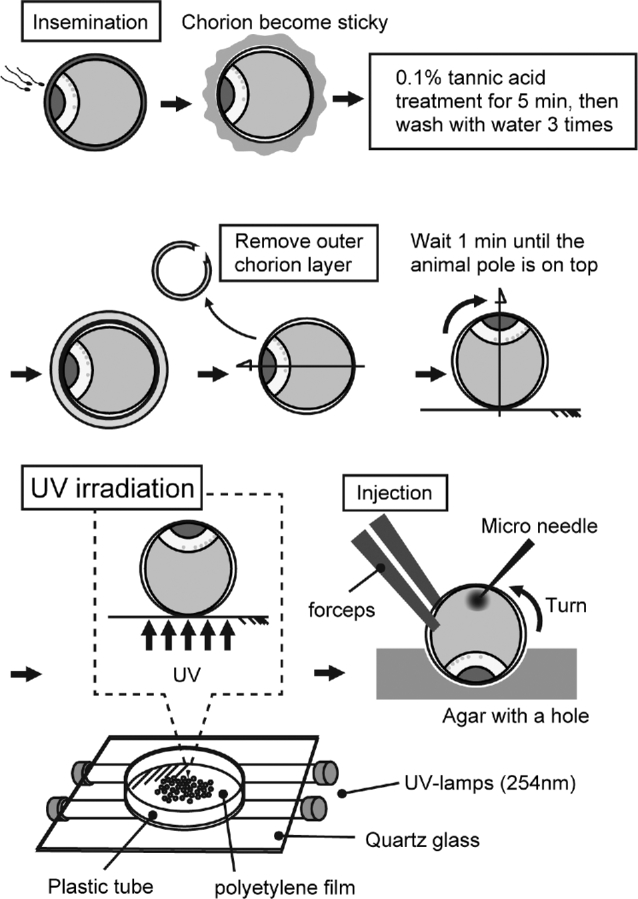
Schematic of UV irradiation of sturgeon embryos.

Gametes from breeding pairs of sterlet (*Acipenser ruthenus*) were collected according to [14] and the eggs were fertilized. Briefly, the fish (3–5 years old, weighing 0.67–1.18 kg) were kept outdoor in 4 m^3^ tanks (water temperature 8°C–12°C) in a hatchery at the Research Institute of Fish Culture and Hydrobiology in Vodnany, Czech Republic. Before spawning, the fish were moved to a closed recirculation system with the water temperature elevated to 15°C within 24 h, and held for 3–4 days without feeding. To induce spermiation, males were given a single intramuscular injection of 40 mg/kg body weight (b.w.) of acetone-dried carp pituitary homogenized extract (CPE). Sperm was collected 42 h after the hormonal injection from the urogenital papilla using a catheter, transferred to a separate cell culture container (250 ml), and stored at 4°C until use. In females, ovulation was induced with two acetone-dried CPE injections, the first with a dose of 0.5 mg/kg b.w. and the second, 12 h later, of 45 mg/kg b.w. The ovulated eggs were collected 42 h after the first injection, and fertilized with the stored sperm in dechlorinated water at 15°C. Fertilized eggs become sticky when they come in contact with water, and the sticky chorion makes it difficult to manipulate and cultivate them. Therefore, the stickiness was removed by treating with 0.1% tannic acid three times for 2 min each, and subsequently washing with filtered water three times. The outer chorion was removed manually using fine forceps under a stereomicroscope (Figure 2). The embryos were kept in dechlorinated tap water (20 embryos per 40 ml) at 15°C, and the water replaced each day until hatching. Upon hatching (approximately 120° days), they were transferred to an aquarium and fed tubifex worms and dried pellets one to three times per day. Some fish were sacrificed using a tricaine overdose and cervical dislocation at 25 days post fertilization (dpf) to count the number of PGCs as described below. We followed Ginsburg and Dettlaff for describing the sterlet embryonic stages [18].

### Ultraviolet irradiation and microinjection

The dechorionated eggs were transferred to a polyethylene film fixed on a plastic ring and placed on a UV transilluminator (UVP) for 1 min in order to let embryos orient animal pole side up (Figure 2). Embryos still facing sideways (rarely) after 1 min were discarded. The transilluminator was equipped with five UV lamps, and a wavelength of 254 nm was used for irradiation. The UV intensity was measured by a UV meter (Ushio), and the dose adjusted for the duration of UV radiation. Three experiments were performed (see Figure 1). The first experiment simply looked at the effect of UV radiation on the germ plasm. For this, three embryos at the one-cell stage were subjected to 360 mJ/cm^2^ of UV radiation, and three more were used as control (without UV radiation). The development of germ plasm in both groups was observed by transmission electron microscopy (TEM). Furthermore, to study germ plasm localization in the embryos after UV irradiation, 10 embryos from this group (and 5 control embryos) were injected with mixture of z*fbuc*-GFP mRNA/Rhodamin-dextran (MW 500,000) at the vegetal pole [19]. The visualized germ plasm was observed at 10, 13, and 16 hours post fertilization (hpf) to monitor the change in germ plasm localization for the surviving embryos from each group.

Experiment 2 had the objective of determining the optimum UV dosage. Four batches of approximately 30 one-cell embryos each were made. While one was used as the control (no UV radiation), the other three were subjected 120, 240, and 360 mJ/cm^2^, respectively, of UV radiation (wavelength = 254 nm) to the vegetal pole. Experiment 3 was done to determine the optimal stage of the embryo for best results. Therefore, three batches of approximately 30 embryos, each at the one-cell, two-cell and four-cell stage, respectively, were subjected to UV radiation at a fixed dose of 360 mJ/cm^2^. As shown in Figure 1, the embryos in experiments 2 and 3 were given an injection of FITC-dextran (MW 500,000) at the vegetal pole to visualize PGCs [19, 20]. Sturgeon PGCs can be visualized in vivo simply by the injecting high molecular weight FITC-dextran into the vegetal pole of one- to four-cell stage embryos [19]. The association between the number of hatching embryos and treatment was tested using the Fisher exact test, and the *P*-values were adjusted using the Benjamini–Hochberg method in order to reduce the false discovery rate for multiple tests (*P* < 0.05), using R software (version 3.4.1).

### Germ plasm and primordial germ cell analysis

GFP-labeled germ plasm in the vegetal pole region at the blastula stage of both control and experimental groups was studied at 10, 13, and 16 hpf, by means of an inverted fluorescence microscope with an imaging system (Olympus IX83 microscope equipped with a Hamamatsu digital camera C10600 ORCA-R2). The distribution of GFP labeled germ plasm both in the control and UV-irradiated embryos was analyzed as follows. Since the *zfbuc*-GFP mRNA injection failed to label the entire germ plasm because of the large molecular weight of the mRNA [14], only regions that could be visualized well were selected for quantifying the germ plasm and studying its distribution. First, a point of contact among three rhodamin-labeled blastomeres near the point of injection was selected and GFP-positive germ plasm was photographed. A circle of radius 250 μm was drawn around this point on the image, and the gross area of GFP-labeled germ plasm in the circle determined using imageJ software (NIH) manually. The gross area of the germ plasm (μm^2^) at the different stages was compared using the Welch test. *P*-value less than 0.05 was considered statistically significant.

The numbers of PGCs visualized by FITC in the UV-irradiated and control embryos were counted at the tailbud stage, pre-hatching stage, and 25 dpf under a fluorescent stereomicroscope (Leica) based on the protocol of [19]. The number of PGCs in each treatment was compared using the Steel-Dwass test by means of the Monte Carlo method using R software (version 3.4.1; NSM3 package). *P*-value of less than 0.05 was considered statistically significant.

### Histology

Embryos at the 16- to 32-cell stage were fixed with 2% glutaraldehyde and 2% PFA in phosphate buffer (PB), rinsed three times with PB, and postfixed with 4% osmium tetroxide for 2 h and again rinsed three times with PB to prepare them for electron microscopy. Samples were dehydrated through an acetone series, and finally embedded in resin (Polybed 812). The embryos were oriented so that sections were cut at the vegetal pole along the animal–vegetal axis. A series of ultrathin sections were cut using a Leica UCT ultramicrotome (Leica, Germany), double stained with uranyl acetate and lead citrate, mounted on a grid, and observed using a transmission electron microscope (JEOL 1010, JEOL Ltd).

## Results

### Decrease of germ plasm in the ultraviolet-irradiated embryos

**Figure 3.**
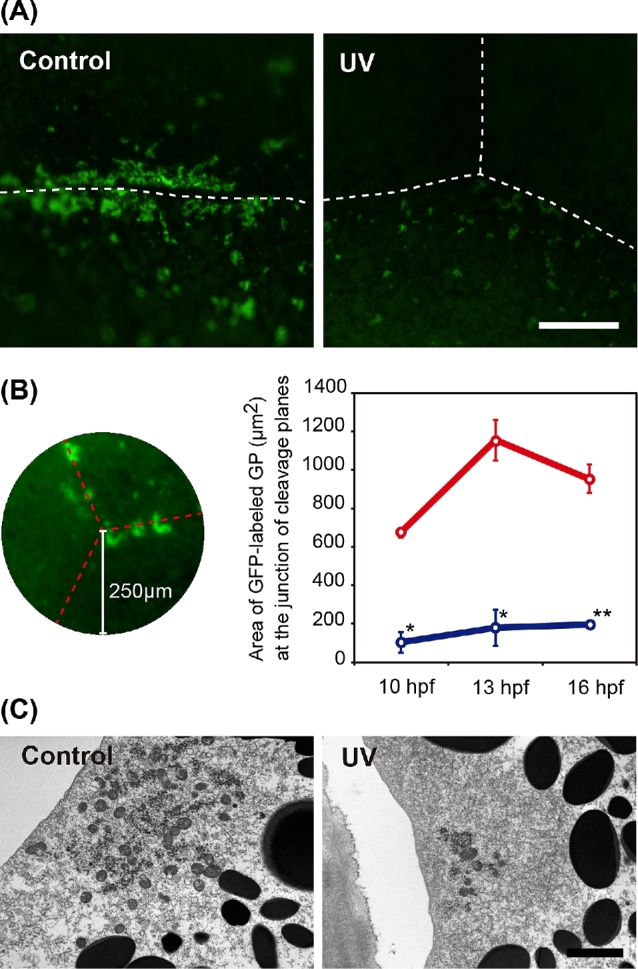
Reduction in germ plasm at the vegetal pole as a result of UV irradiation. (A) Control: GFP-labeled germ plasm at the cleavage furrow at blastula stage in a control embryo. The germ plasm forms accumulated large islands on the cleavage furrow. UV: The germ plasm at the cleavage furrows in a UV-irradiated embryo. The islands are sparse and scarce when compared to the control. (B) Area selected for quantifying the germ plasm. Left: A point of contact among three blastomeres was selected, and a circle of radius 250 μm was drawn around the point. Broken lines indicate cleavage furrows. The gross area of GFP germ plasm was measured. Right: Comparison of the gross area of GFP germ plasm between control (upper) and UV embryo (lower) at each stage. (C) TEM image of the germ plasm. Scale bar in A and C indicates 50 μm and 2 μm, respectively. Means with asterisk(s) are significantly different when compared to the control at same stage (*P* < 0.001*, *P* < 0.01**).

In the UV-treated group, islands of germ plasm labeled with zfbuc-GFP were seen as small particles at the cleavage furrows in the vegetal pole during cleavage (10–16 hpf). In contrast, germ plasm accumulated abundantly along the cleavage furrows in the control embryos (Figure 3A). The gross area of germ plasm at any given position (e.g. Figure 3B, left) in the irradiated group was at least five times smaller than the corresponding region in the control at each stage (10–16 hpf) (Figure 3B, right). This was further confirmed by means of TEM (Figure 3C). Furthermore, fewer mitochondria were observed around the germ plasm in the irradiated embryos (Figure 3C).

### Ultraviolet irradiation eliminates primordial germ cells

**Figure 4.**
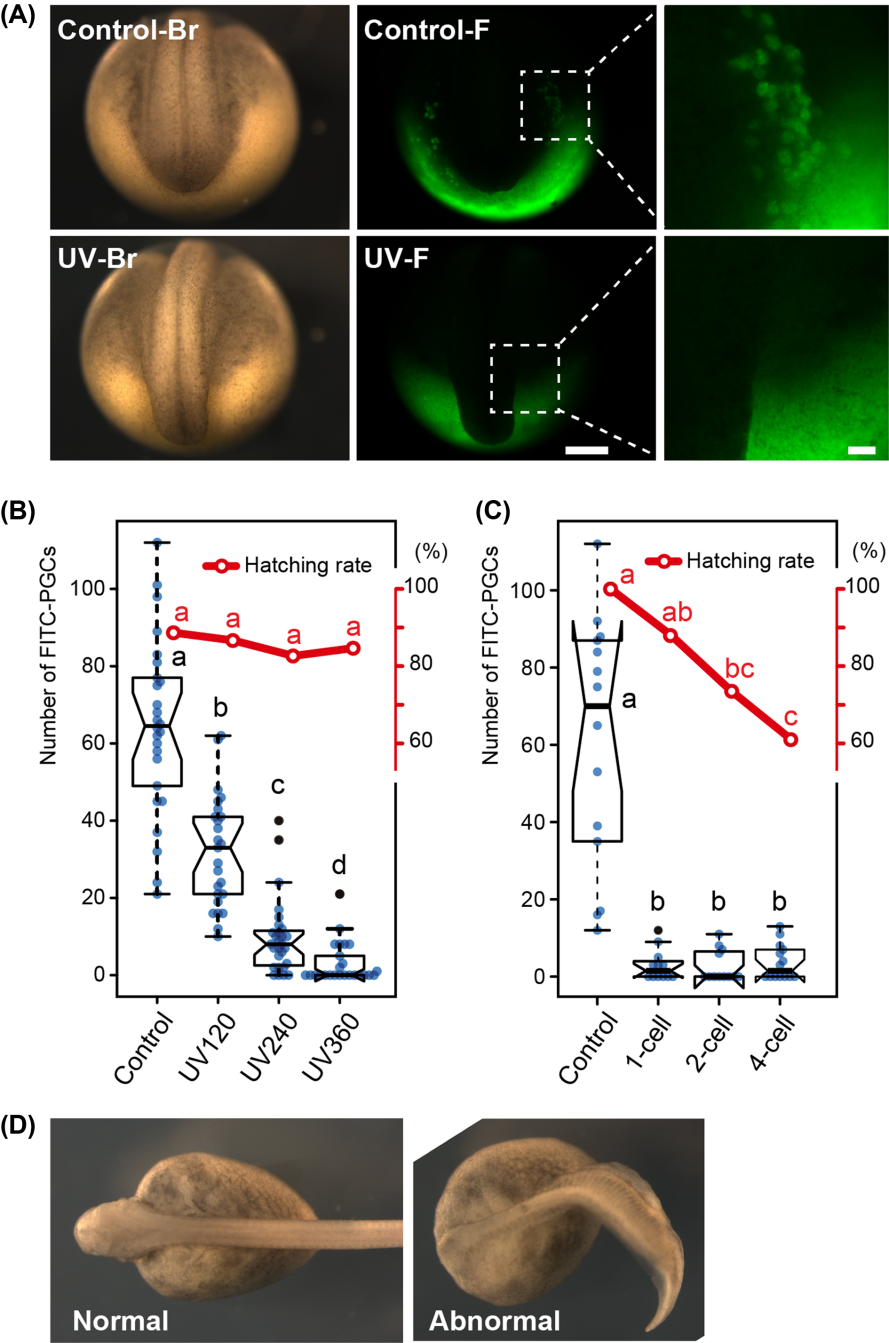
Reduction in the number of PGCs at the neurula to tail bud stage after UV irradiation. (A) Control and UV irradiate embryos with FITC-dextran labeled PGCs at the tail bud stage [19]. The UV-irradiated embryo (second row) lacks FITC-PGCs around the surrounding region of the developing tail, while the control embryo (first row) has many. Br: bright field view. F: fluorescence view. The last column shows the magnified image of the boxes in the middle columns. (B) The number of PGCs declined as the UV intensity increased (box plots), whereas the hatching rate in the UV-irradiated group was same with that of control (line graph). UV irradiation was performed on the one-cell stage embryos. (C) The drastic reduction in the number of PGCs even in embryos irradiated at the four-cell stage at the intensity of 360 mJ/cm^2^ (box plots), although the hatching rate decreased with the developing embryos (line graphs). (D) The nonirradiated control and abnormal embryos at the four-cell stage after receiving UV irradiation at a dose of 360 mJ/cm^2^. Box plots: center lines show the medians; box limits indicate the interquartile range (between the 25th and 75th percentiles); whiskers extend 1.5 times past the interquartile range, outliers are represented by black dots; data points by blue dots. n = 25, 28, 25, 26 sample points in B and n = 10, 5, 13, and 11 in (C). Scale bars in (A) indicate 500 μm (middle column) and 100 μm (right column), respectively. Boxes and points with different letters signify significant difference (*P* < 0.05).

The effect of UV irradiation on PGC development was studied by counting the number of FITC-labeled PGCs in each experimental group at the neurula stage (Figure 4A). It declined significantly as the intensity of UV irradiation increased (Figure 4B). In the group exposed to 360 mJ/cm^2^ UV irradiation (UV360), although a few ectopic FITC-labeled PGCs were seen in some embryos (Figure 4B, Table 1), most embryos were completely devoid of PGCs in the surrounding region of the tail bud, the expected area of accumulation (Figure 4A).

**Table 1.** Summary of PGC frequencies in each treatment.

Treatment	Stage	N	Number of PGCs in each embryo/larva	Range	Average	SD	P*
Control	Neurula	26	21, 24, 32, 37, 45, 45, 49, 56, 58, 60, 62, 63, 64, 65, 66, 68, 70, 75, 76, 77, 81, 83, 89, 98, 101, 112	21–112	64.50	22.68	0.027
	25 dpf	10	58, 65, 70, 71, 83, 85, 87, 88, 124, 136	58–136	86.70	25.08	
UV120	Neurula	25	10, 12, 16, 16, 16, 19, 21, 21, 23, 24, 27, 29, 33, 34, 35, 38, 40, 41, 41, 43, 45, 46, 48, 61, 62	10–62	32.04	14.43	0.361
	25 dpf	5	7, 15, 17, 20, 56	7–56	23.00	19.07	
UV240	Neurula	28	0, 0, 0, 0, 2, 2, 2, 3, 5, 6, 7, 7, 7, 8, 8, 9, 10, 10, 10, 11, 11, 12, 13, 15, 17, 24, 35, 40	0–40	9.79	9.67	0.548
	25 dpf	13	0, 0, 1, 4, 4, 5, 10, 12, 14, 17, 17, 24, 55	0–55	12.54	14.81	
UV360	Neurula	25	0, 0, 0, 0, 0, 0, 0, 0, 0, 0, 0, 0, 0, 0, 0, 1, 2, 3, 5, 8, 8, 8, 8, 12, 21	0–21	3.04	5.20	0.036
	25 dpf	11	0, 0, 0, 0, 0, 0, 0, 0, 2, 2, 3	0–3	0.64	1.12	

*Welch t-test.

Hatching rate was similar between the UV-irradiated and control groups when irradiation was done at the one-cell stage (Figure 4B). All embryos developed normally after any dose in the experimental range of UV irradiation (UV120–UV360), and grew up as healthy controls until sacrificing (25 dpf). The rate of PGC elimination, which happened only in the treatment group, was similar across the one to four-cell stages, although the survival rate declined as the irradiation was done at later stages (Figure 4C). Some embryos, which were irradiated at the two- to four-cell stage, showed malformations such as truncated and bent/twisted body shape during embryonic development (Figure 4D). These embryos did not hatch and eventually died in the chorion (Figure 4C).

**Figure 5.**
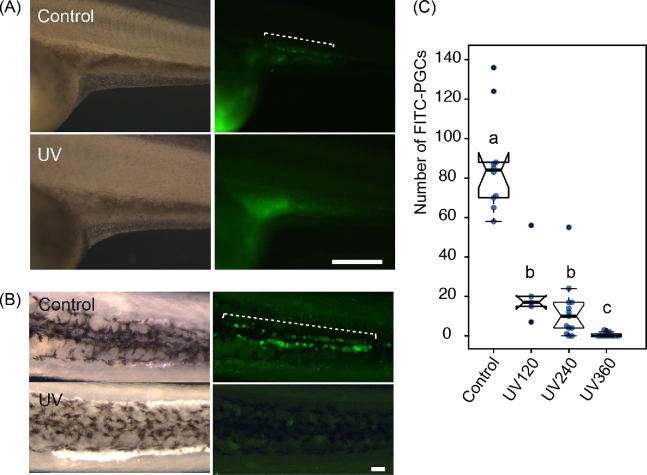
The continued absence of PGCs in the UV-irradiated fry at 25 days post fertilization (dpf). (A) The gonadal region of the control and UV-irradiated embryos at the stage 32. The right column shows corresponding fluorescent images of those in the left column. The UV-irradiated embryo completely lacks FITC-PGCs, while the control embryo has many FITC-PGCs in the gonadal region (broken line). (B) Ventral view of FITC-PGCs in the upper parts of the abdominal cavity after removal of intestines at 25 dpf. The broken line in the fluorescent view shows FITC-PGCs in the control, whereas the UV-irradiated embryo completely lacks FITC-PGCs. (C) The number of PGCs per fry in each group at 25 dpf. Center lines show the medians; box limits indicate the interquartile range (between the 25th and 75th percentiles); whiskers extend 1.5 times beyond the interquartile range, outliers are represented by black dots; data points are plotted as blue dots. n = 10, 5, 13, 11 sample points. Scale bars in (A) and (B) indicate 500 μm and 100 μm, respectively. Bars with different letters are significantly different (*P* < 0.05).

The number of PGCs in the larvae from experiment 2 was counted at 25 dpf. FITC-labeled PGCs rarely appeared in the genital ridges of UV-irradiated embryos (UV360) at later stages: pre-hatching stage and 25 dpf (Figure 5A and B). Three embryos out of 12 had small numbers of FITC-labeled PGCs (2, 2, and 3) in the UV360-irradiated group (Figure 5C, Table 1). Although the number of PGCs in the controls at 25 dpf increased significantly when compared to those in the neurula stage, the number of remaining PGCs in the UV360-irradiated 25 dpf larvae was significantly lower than in the neurula stage (Welch test, *P* < 0.036).

## Discussion

This study was undertaken to determine if UV irradiation is a viable technique for sterilizing fish embryos of the sterlet (*Acipenser ruthenus*) that can then be used as a surrogate and receive GSCs from an endangered target sturgeon species, in an effort to rescue the latter species from extinction. We found that the amount of germ plasm (labeled with *zfbuc*-GFP mRNA) in the sterlet declined drastically after the vegetal pole of the embryo was subjected to UV irradiation, when compared to the control sterlet embryos that received no UV irradiation. Visualization by TEM revealed that the size of germ plasm at the cortical region became smaller, and the number of mitochondria around the germ plasm declined in the irradiated embryos. These results indicate that UV irradiation destroyed and dispersed the germ plasm complex including mitochondria. Fragmented germ plasm and a high rate of vacuolation in mitochondria as a result of UV irradiation have also been seen in Stage-6 Xenopus eggs [21]. It has been shown in anurans that germ plasm particles in a wide area of the oocyte undergo cytoskeletal-dependent aggregation toward cleavage planes to form large islands at cleavage furrows during early cleavage stages [22]. Although we did not ascertain any damage to the cytoskeletal structure in our embryos, it is possible that it was also damaged by the UV irradiation, and that as a result, the germ plasm failed to aggregate at the cleavage furrows. Although the mechanism is speculative, it is clear that the accumulation of germ plasm, Buc-GFP labeled nuage, and mitochondria cloud are inhibited as a result of UV irradiation in sturgeon embryos.

The hatching rate of embryos in the UV-irradiated group was the same as that of the control when the irradiation was done at the one-cell stage. The developmental fate of yolk blastomeres at the vegetal hemisphere of sturgeon egg is extraembryonic nutrition; they are surrounded by endoderm cells and digested as the embryo develops. Therefore, yolk blastomeres damaged by UV irradiation are unlikely to impede embryonic development. On the other hand, some embryos to which UV irradiation was applied at the later stages of development (two- to four-cell stage) were malformed and perhaps as a result, did not hatch. In *Xenopus*, it has been shown that UV irradiation disrupts the formation of an array of parallel microtubules associated with the dorsal axis formation [23]. It appears that UV irradiation in the later stages of sturgeon embryos resulted in this sort of morphogenetic disorder in this study. Therefore, UV irradiation at the one-cell stage is expected to be the proper treatment for sterilizing sterlet embryos.

We found some irradiated embryos with a few FITC-positive PGCs in the neurula stage embryos and 25 dpf fry. There are two possible explanations for this: (1) there were physiological or morphological differences against UV irradiation among embryos, and (2) the intensity of applied UV irradiation was not applied uniformly to all embryos, perhaps because of the limitations of the experimental conditions. Favoring the former explanation are any systematic or even stochastic differences among the embryos in chorion thickness, amount of germ plasm in an embryo, and position/depth of germ plasm. In *Xenopus*, after centrifugation treatment on eggs, the germ plasm locates much deeper in the cytoplasm, and PGCs are formed even after sufficient dose of UV irradiation in the treated embryos [17]. It has been suggested that the amount of germ plasm supplied maternally and number of PGCs in embryos vary among embryos in some fish species [24,25]. Sturgeon embryos also vary in the number of PGCs as in the other species [14,19]. Thus, slight differences in the topology and/or amount of the germ plasm can affect the efficiency of PGC elimination by UV. Indeed, about 70% of the UV is absorbed within the thin layer (60 μm) of the egg surface in *Xenopus* [26]. However, since we used a transilluminator designed for DNA analysis (in which five lamps were placed at intervals of about 3 cm) to irradiate eggs in this study, we cannot exclude the possibility of uneven UV irradiation of the embryos. On the other hand, the hatching rate of embryos was high after UV irradiation even at the highest dose. Clearly, the procedure for UV irradiation needs to be optimized for future experiments.

There are some problems to be solved before using UV-irradiated sturgeon embryos for the production of surrogate hosts. In the present research, we observed their healthy development after UV treatment until 25 dpf. However, to ensure the success of gamete production via surrogate host, it is important to check the development of UV-treated sturgeons until the adult stage. It is also necessary to test if complete gonads can be formed in both sexes. In medaka and zebrafish, complete removal of PGCs by injection of antisense MO against the *dead end* (*dnd*) gene leads to the production of an all-male population in the treated embryos [27,28], although in loach, *Misgurnus anguillicaudatus*, and goldfish, *Carassius auratus*, sterilized embryos develop into both sexes [29,30]. Sterilization of sturgeons has also been achieved using same app roach (knock-down of *dnd* gene by MO), but sex differentiation after the removal of PGCs is still unclear [7]. If sex determination is biased after sterilization treatment in sturgeon, the surrogate production will obviously be affected; sex reversal treatment will then be needed to produce chimeras with both sexes. As expected, the number of PGCs increased in nonirradiated controls in the present study. However, although there was a significant reduction from the neurula stage in the UV360 group, a few PGCs still remained in some larvae at 25 dpf. Therefore, it is important to study the fate of the remaining PGCs in these embryos after UV irradiation. Host-derived sperm or egg generation is not permitted in the surrogate production technique in order to avoid the possibility of hybrids among the offspring and the consequent genetic contamination of the target species. Interspecific hybrids of distantly related species, when viable, are generally sterile due to complications in chromosomal pairing [31]. However, sturgeon hybrids from several species crosses tend to be not just viable but also fertile, even between species with different ploidy levels [32]. Therefore, 100% sterilization is particularly important in the sturgeon surrogate production system.

Despite some shortcomings, however, we believe that our preliminary results show that UV irradiation has the potential to be used for the mass production of PGC-free sterlet sturgeons that can then be used as surrogates for the production of other, endangered, target sturgeon species. Our continued work in this field has served to show that surrogate production in sturgeon is rapidly becoming feasible [3,6,7,14,19]. We believe that his technique for PGC elimination will be an invaluable tool for surrogate production of endangered sturgeon.

## Notes

Edited by Dr. Kyle Orwig, PhD, University of Pittsburgh
